# Reply to Comment Clement *et al.*: (Prevalence of Antibodies against Hantaviruses in Serum and Saliva of Adults Living or Working on Farms in Yorkshire, United Kingdom)

**DOI:** 10.3390/v6093425

**Published:** 2014-09-12

**Authors:** Lisa J. Jameson, Autilia Newton, Louise Coole, Edmund N. C. Newman, Miles W. Carroll, Nick J. Beeching, Roger Hewson, Robert M. Christley

**Affiliations:** 1 Microbiology Services, Public Health England, Porton Down, Wiltshire SP4 0JG, UK; E-Mails: edmund.newman@phe.gov.uk (E.N.C.N.); miles.carroll@phe.gov.uk (M.W.C.); roger.hewson@phe.gov.uk (R.H.); 2 Institute of Infection and Global Health, University of Liverpool, Liverpool L69 7BE, UK; E-Mail: robc@liverpool.ac.uk; 3 Public Health England, Yorkshire and the Humber, York YO41 1LZ, UK; E-Mail: autilia.newton@phe.gov.uk; 4 Field Epidemiology Services, Public Health England, Leeds LS2 7UE, UK; E-Mail: louise.coole@phe.gov.uk; 5 Clinical Sciences, Liverpool School of Tropical Medicine, Liverpool L3 5QA, UK; E-Mail: beeching@liverpool.ac.uk

We acknowledge Clement and colleagues for their comments [1] on our paper [2]. We agree that many controversies are being discussed by the hantavirus community, particularly surrounding the interpretation of serological results and the designation of new species and strains. Within this setting, we are grateful for the opportunity to respond to the key factual and methodological points raised by Clements *et al.*

The decision of whether Saaremaa virus (SAAV) is a separate species of hantavirus is not within the scope of our study: we followed the guidelines of the International Committee on Taxonomy of Viruses (ICTV) which lists SAAV separately from Dobrava virus. Serological cross-reactions between certain hantavirus species are widely acknowledged and referred to in our discussion of results. Given the published molecular evidence of a Seoul hantavirus in the region [3,4] and unpublished confirmation of virus amplified from a human sample with 100% homology to Cherwell [5], we are confident in assuming that the samples reactive for both Seoul and Hantaan represent Seoul infection.

We did not intend to suggest that insectivore-related hantaviruses are pathogenic to humans and our text does not state this. It does assume that transmission occurs (even if only between reservoir animals) through excreted virus, which is the known route for all studied hantaviruses. Transovarial transmission is not an effective route for maintenance of hantaviruses, so excretion seems the most likely manner for insectivore-borne hantaviruses to be sustained in nature.

We acknowledge that it might have been useful for readers less familiar with the history of hantavirus research in the UK if we had cited the work of McKenna *et al.* [6]. However, that study focused on symptomatic patients and ours used a subset of healthy volunteers, limiting the validity of direct comparison. We believe that the PHLS farmers cohort study [7] provides a more appropriate comparator group. Regarding the inclusion of IgM titres, it is unreasonable to compare high titre acute samples, as reported by McKenna *et al.* [6], Jameson *et al.* [3] and Taori *et al.* [8], to samples from persons who may have been exposed decades prior to sampling. Given that our volunteers were not sick, we are unable to see the benefit of testing for IgM in our anonymised study.

The principal aim of our study was to provide a preliminary investigation of the seroprevalence to hantaviruses among farm workers and dwellers in a region of Great Britain in which a recent case of HFRS had occurred. As part of this investigation, a limited questionnaire was answered by participants to explore some possible risk factors. In order to limit the burden to participants, the questionnaire was deliberately kept short and only a limited set of questions was included. The results only pertain to the study sample (*i.e.*, farmers and farm dwellers) and the potential impact of using a convenience sample, rather than a random sample, is noted in our discussion. Further studies to evaluate the role of a wider range of potential risk factors are now warranted.

Within Table 2, “NA” is used to denote “not available” (rather than not assessed). Several odds ratios were not available, as the calculations involved category values of zero; for example, all 9 seropositive participants reported seeing rats and none reported not seeing rats.

The commercially available Euroimmun IFA (Luebeck, Germany) is a useful and convenient diagnostic assay for acute samples and serosurveys as it covers a panel of hantavirus species. It is not designed to confirm the causative species. We agree with Clement *et al.* that only molecular methods are able to do this. Thus we were cautious in interpreting the results of the samples reacting to the other hantavirus species and at no point suggested they were likely to be result of a Sin Nombre (SNV) infection, instead we described them as SNV/Puumala-like hantaviruses. We and peer reviewers at the time understood that this subtle statement alluded to the inexactness of the IFA technique at this level. We focused attention on the results of SEOV, since this is where we had provided molecular evidence of its existence, as indicated above.

While acknowledging the concerns of Clements *et al.*, we stand by the key findings and design of our study, which has provided new data on the seroprevalence of hantavirus infection in a farming community in which the first molecularly confirmed case in the UK had recently been detected [2]. More detailed studies logically follow on from these preliminary findings and are now in progress.
